# Von akutem Koronarsyndrom bis Zoster: Differenzialdiagnostik bei segmentaler und somatischer Dysfunktion an Brustwirbelsäule und Rippen

**DOI:** 10.1007/s00132-022-04227-8

**Published:** 2022-03-10

**Authors:** Hein Schnell

**Affiliations:** Praxis für Orthopädie und Unfallchirurgie, Baldestr. 8, 80469 München, Deutschland

**Keywords:** Vegetatives Nervensystem, Brustschmerzen, Differentialdiagnostik, Viszero-vertebraler Reflex, Blockierung, Autonomic nervous system, Chest pain, Differential diagnostics, Viscero-vertebral reflex, Sympathetic trunk

## Abstract

**Hintergrund:**

Segmentale und somatische Dysfunktionen im Thorakalbereich können zu unterschiedlichen klinischen Bildern führen. Es gilt, drei wesentliche Varianten zu unterscheiden.

**Klinik:**

1. Lokale Schmerzen

Hierbei müssen auch potenziell lebensbedrohliche Differenzialdiagnosen bedacht und im Zweifel die Chest-Pain-Notfalldiagnostik initiiert werden.

2. Vertebro-viszeraler Reflex

Der sympathische Grenzstrang hat seine segmentalen Ursprünge im Wesentlichen in den thorakalen Segmenten, dies führt zu einer hochgradigen neuronalen Vernetzung mit den Thorax- und Oberbauchorganen. „Unspezifische“ Thorax- und Bauchbeschwerden können deswegen ihre Ursache in segmentalen und somatischen Dysfunktionen der thorakalen Segmente haben.

3. Viszero-vertebraler Reflex

Viszerale Noziafferenzen gelangen über die vegetative Vernetzung zu thorakalen Segmenten und können hier zu schmerzhaften thorakalen Dysfunktionen führen. Diese können erste Anzeichen einer ernsthaften strukturellen Erkrankung, z. B. Neoplasie oder Ulkus, der Thorax- und Oberbauchorgane darstellen.

**Differenzialdiagnostik:**

Die Differenzialdiagnostik ist anspruchsvoll, die manuelle Medizin kann einen entscheidenden Beitrag leisten. Die biomechanischen und neurophysiologischen Besonderheiten am Thorax müssen dafür bekannt sein.

## Abstract (en)

**Background:**

Segmental and somatic dysfunction in the thoracic section can lead to various clinical symptoms. It is necessary to distinguish three variants.

**Clinical picture:**

1. Local pain

Potentially life-threatening differential diagnoses have to be considered, and when in doubt chest pain emergency diagnostics must be initiated.

2. Vertebro-visceral reflex

The main segmental roots of the sympathetic trunk are in thoracic segments, this results in a high-grade linking to thoracic and abdominal organs. “Non-specific” thoracic and abdominal symptoms can be caused by segmental and somatic dysfunction in thoracic segments.

3. Viscero-vertebral reflex

Visceral nociception is transmitted via vegetative fibers to thoracic segments. Here, painful dysfunction can occur, which might be the first sign of severe structural disease like neoplasia or ulcer in thoracic or abdominal organs.

**Differential diagnostics:**

Differential diagnostics is challenging, and manual medicine can contribute substantially. Biomechanical and neurophysiologic particularities must be known.

Segmentale und somatische Dysfunktionen im Thorakalbereich können facettenreich in Erscheinung treten. Beim Leitsymptom „Thoraxschmerz“ müssen lebensbedrohliche Differenzialdiagnosen ausgeschlossen werden. Über den sympathischen Grenzstrang besteht eine enge neuronale Vernetzung mit den Thorax- und Oberbauchorganen. „Unspezifische“ Thorax- und Bauchbeschwerden können ihre Ursache in segmentalen und somatischen Dysfunktionen der thorakalen Segmente haben. Schmerzhafte thorakale Dysfunktionen können erste Zeichen einer ernsthaften strukturellen Erkrankung der Thorax- und Oberbauchorgane sein.

## Einleitung

Segmentale und somatische Dysfunktionen entstehen im Thorakalbereich auf dem Boden der gleichen Mechanismen wie an den übrigen Wirbelsäulenabschnitten. Auch hier gilt das Prinzip der Konvergenz auf segmentaler Ebene sowie der grundlegende Pathomechanismus der segmentalen und somatischen Dysfunktion mit einer Noziafferenz und (schutz‑)reflektorischer motorischer Antwort. In diesem Kontext sind am Thorax zwei (neuro‑)anatomische Aspekte für die klinische Symptomatik, die Diagnostik und Therapie von Bedeutung:

- Das muskuloskelettale System am Thorax ist klar segmental gegliedert (z. B. Costa 5 entspricht Segment Th 5). Die Symptome am muskuloskelettalen System bleiben weitgehend auf den Thorax begrenzt (z. B. Interkostalneuralgie). Eine segmentale und somatische Dysfunktion thorakaler Segmente kann auch an den Rippen klinisch apparent werden. Die Rippenfunktion kann sehr gut mittels MIP-Algorithmus untersucht werden und die Rippen können sehr selektiv direkt manuell behandelt werden. [1, 4].
- Der sympathische Teil des vegetativen Nervensystems ist mit seinen thorakal gelegenen Ursprungsneuronen und dem Grenzstrang wesentliches Zentrum vegetativ-sympathischer Innervation der Thorax- und Bauchorgane. Neuroanatomisch besteht eine hochgradige Vernetzung von somatischem und vegetativ-sympathischem Nervensystem [2, 9, 18, 21]. Dies führt zu facettenreichen klinischen Bildern.

## Klinisches Bild der segmentalen und somatischen Dysfunktion an BWS und Rippen

Isoliert betrachtet ist die segmentale und somatische Dysfunktion an BWS und Rippen vergleichsweise harmlos sofern es lediglich zu lokalen Schmerzen kommt. Herausfordernd ist die Komplexität der klinischen Symptome, die auf die oben dargestellten neurophysiologischen und vegetativen Aspekte zurückzuführen sind.

Beim Leitsymptom Thoraxschmerz an potenziell lebensbedrohliche Erkrankungen denken

**Figure 1:**
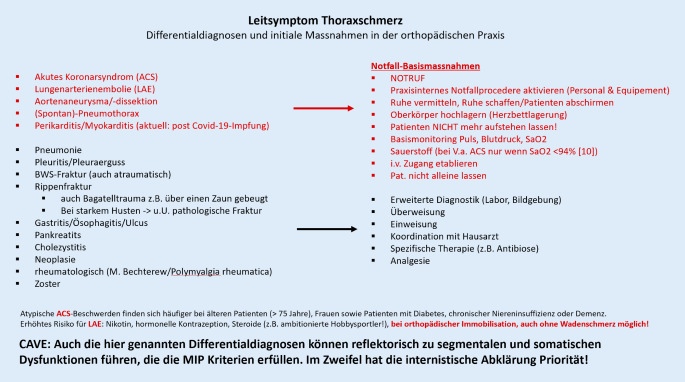

Zu unterscheiden sind im Wesentlichen drei Erscheinungsformen:

- Lokale bis regionale Schmerzen mit gürtelförmiger Ausstrahlung entlang der Rippen (segmental). *Cave:* Beim Leitsymptom Thoraxschmerz müssen die potenziell lebensbedrohlichen Erkrankungen der Thorax- und Bauchorgane in die differenzialdiagnostischen Überlegungen immer mit einbezogen werden. Im Zweifel gilt es immer, eine adäquate Notfalldiagnostik mit aller Konsequenz zu vertreten und einzuleiten (d. h. praxisinternes Notfallprozedere aktivieren – Rettungsdienst/Notarzt – Einweisung in Notfallzentrum idealerweise mit Chest-Pain-Unit) [8]. Zu den wichtigsten Differenzialdiagnosen und Notfallmaßnahmen siehe Abb. 1. Nach Ausschluss lebensbedrohlicher Differenzialdiagnosen kann die Behandlung von segmentalen und somatischen Dysfunktionen eine äußerst befreiende Wirkung haben. Die „harmlose“ Diagnose der Dysfunktion reduziert Ängste bei den Patienten und verhindert das Psycho-Etikett.
- Schmerzarme oder gar schmerzfreie segmentale und somatische Dysfunktionen der thorakalen Segmente können über die sympathischen Efferenzen zu Fehlsteuerung der Organfunktionen führen [9, 15]. Es resultieren Beschwerden, für die anderweitig keine Ursache gefunden werden kann, meist werden sie als „unspezifisch“ oder „funktionell“ betitelt. Funktionell ist an dieser Stelle meist ein Euphemismus für psychogen, wenngleich der Funktion tatsächlich eine wesentliche Bedeutung zukommt und mit der segmentalen und somatischen Dysfunktion eine spezifische Ursache gegeben ist. Die manualmedizinische Behandlung und Auflösen der Dysfunktion kann die Organsymptomatik verbessern.
- Chronisch rezidivierende schmerzhafte Dysfunktionen an BWS und Rippen, die sich nach primär erfolgreicher manualmedizinischer Behandlung immer nur kurz bessern und schnell rezidivieren, können ihre Ursache in anhaltendem nozizeptivem Input aus den inneren Organen haben, die über die afferenten Fasern des vegetativen Systems segmental aufgenommen werden. Auslöser können schwerwiegende strukturelle Erkrankungen sein, beispielsweise Ulzera oder Neoplasien. Abb. 5a–c und Tab. 1 zeigen orientierend, welche Rückenmarksegmente welchen Organen zugeordnet werden können. Hieraus können sich Hinweise ergeben, welche weitere Untersuchung indiziert werden sollte [9, 21].

**Table 1:** 

Organ	Segment
Herz	T1–5
Magen	T5–9
Leber und Gallenblase	T6–9
Pankreas	T5–11
Dünndarm	T9–11
Kolon und Rektum	T8-L2
Niere und Harnleiter	T10-L1
Blase	T10-L1
Ovarien und Eileiter	T9–10
Hoden und Nebenhoden	T9–10, L1–2
Uterus	T10-L1
Prostata	L1–2

## Sympathikus und vegetative Vernetzung

**Figure 2:**
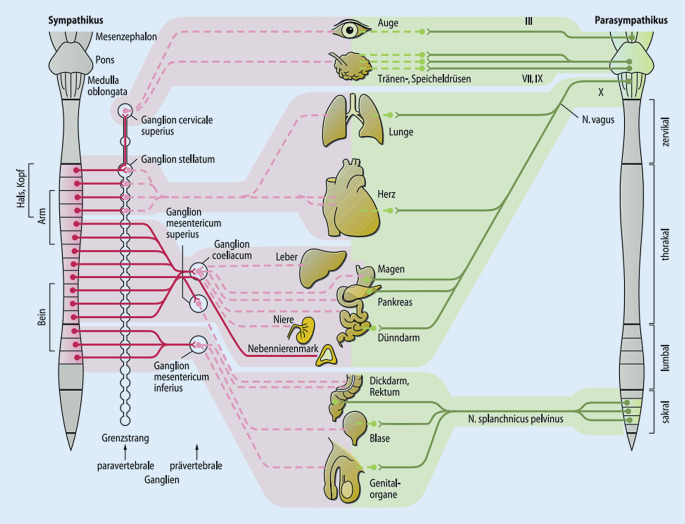

Der sympathische Grenzstrang liegt paravertebral entlang der gesamten Wirbelsäule. Von den thorakalen Segmenten kommt der größte Anteil an sympathischen Efferenzen, die die Thorax- und Bauchorganen viszeroefferent versorgen (Abb. 2). Da die Fasern nach Verlassen der segmentalen Ganglien vielfache, netzartige Verbindungen haben, geht die klare segmentale Zuordnung teilweise verloren.

**Figure 3:**
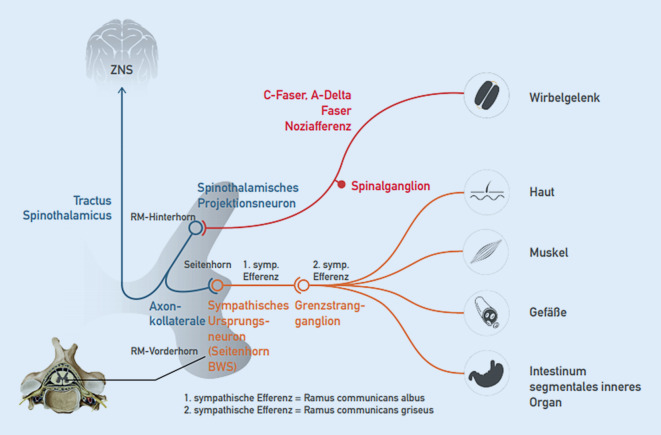

Lange Zeit ging man davon aus, dass die Fasern vom Grenzstrang reine sympathische Efferenzen seien. Mittlerweile ist belegt, dass auch afferente Signale über diese Fasern vermittelt werden [19]. Somit kann auf diesem Weg auch eine viszerale Nozizeption transportiert werden und im Sinne der segmentalen Konvergenz und des Schutzreflexgeschehens zu einer segmentalen und somatischen Dysfunktion von BWS und Rippen führen, welche palpatorisch erfasst werden kann (MIP-Diagnostik). Die direkte Verschaltung der sympathischen Efferenzen auf segmentaler Ebene bezogen auf das Reflexgeschehen bei der segmentalen und somatischen Dysfunktion zeigt Abb. 3.

Die Funktion thorakaler Segmente und das sympathische System beeinflussen sich gegenseitig

Das sympathische Ursprungsneuron (1. Neuron) liegt im Seitenhorn und erreicht über die Vorderwurzel via Ramus communicans albus den Grenzstrang. Hier erfolgt teilweise eine Umschaltung auf ein 2. Neuron, teilweise werden die Fasern zu anderen prävertebral und organnah gelegenen Ganglien geleitet [19]. Das 1. Neuron wird über Axonkollateralen vom spinothalamischen Projektionsneuron gespeist (Abb. 3). Eintreffende Noziafferenzen am spinothalamischen Projektionsneuron können folglich auch die sympathische Aktivität modulieren.

## Polysegmentale Vernetzung sympathischer Innervation

Die sympathischen Efferenzen haben ebenfalls einen segmentalen Ursprung, der größte Anteil im Bereich der BWS (Abb. 2 und Tab. 1). Die einzelnen Grenzstrangganglien werden jedoch aus mehreren Segmenten gespeist [16] und konfluieren im Verlauf zu ihren Zielorganen (Abb. 2). Im klinischen Bild ist deswegen oft kein eindeutiger Segmentbezug mehr erkennbar.

**Figure 4:**
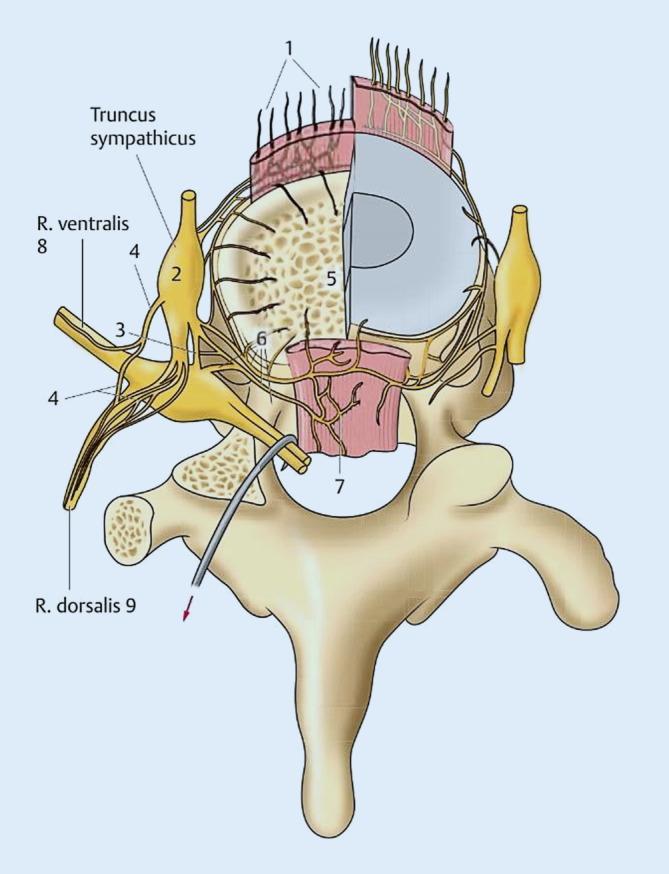

Spinalnerv und Grenzstrang sind zusätzlich hochgradig mit den Afferenzen des vertebralen Bewegungssegments vernetzt (Abb. 4). Ergänzend kommt hinzu, dass die Afferenzen der Strukturen des Bewegungssegments (Diskus, Facettengelenke, Längsbänder) polysegmental über den Grenzstrang laufen [2, 14].

**Figure 5:**
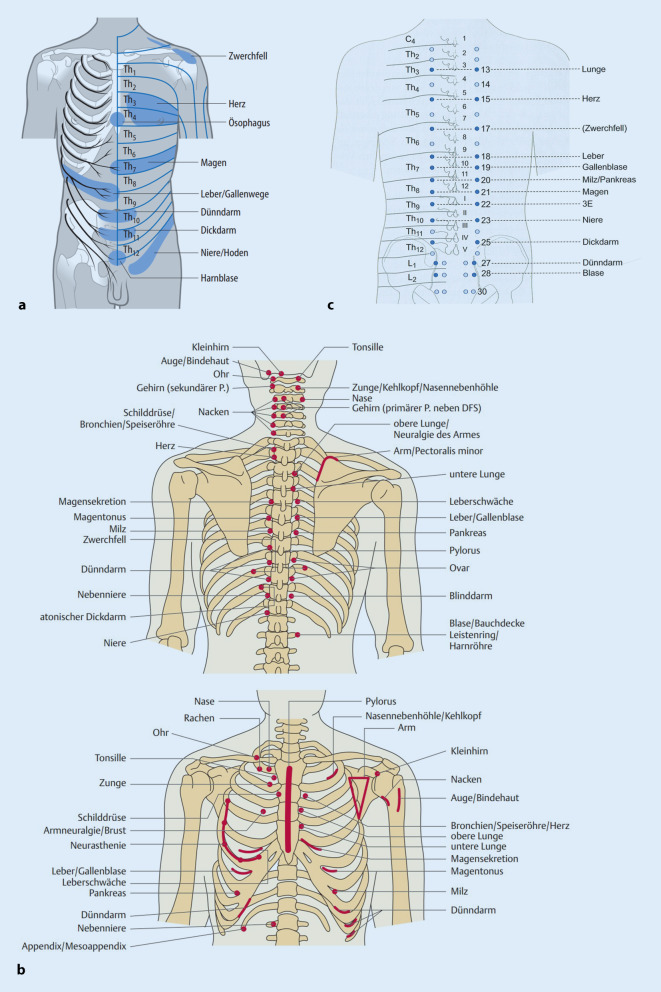

Parallel zu den sympathischen Efferenzen zu den Organen, laufen auch Afferenzen, die z. B. an der Schmerzübertragung aus den Eingeweiden beteiligt sind, zurück zum Rückenmark. Dies findet sein Abbild in den weithin bekannten Head-Zonen (Abb. 5a) [19, 22]. Sehr ähnlich erscheinen die Chapman-Punkte (Abb. 5b; [9, 13]) und auch einige Akupunkturpunkte (Shu-Punkte der Thorax- und Bauchorgane) (Abb. 5c; [20]). Letztlich sind diese Beobachtungen auf die segmentale Vernetzung zurückzuführen. All diese Punkte sind empirisch überliefert und können hinweisend auf einen viszerosomatischen Zusammenhang sein, sind jedoch nicht beweisend. Gleichzeitig stellen sie einen möglichen therapeutischen Zugang dar [5].

Für den klinischen Alltag stellt Tab. 1 orientierend dar, in welchem Rückenmarksegment die präganglionären sympathischen Neurone eines inneren Organs liegen, bzw. welche Organe welchem Rückenmarksegment sympathisch zugeordnet werden können [21], ähnliche Tabellen finden sich bei vielen Autoren, jedoch mit etlichen Abweichungen.

## Manualmedizinische Diagnostik und Therapie am Thorax

### MIP-Diagnostik an Wirbelsäule und Rippen

An den Brustwirbelkörpern kann die MIP-Diagnostik analog zu HWS und LWS durchgeführt werden [1, 4]. Die Rippen sind einer palpatorischen Diagnostik sehr gut zugänglich, eine Dysfunktion der Rippen kann analog zu Wirbelsäulensegmenten ebenfalls mittels MIP-Diagnostik (siehe Leitartikel) diagnostiziert werden. Gesucht wird auch hier die Befundtrias: Hypomobilität, Irritation und Provokation [1, 4, 11]:

**M**obility: Bereits die Inspektion der Atemexkursionen kann einen guten Hinweis auf eine eingeschränkte Mobilität von Rippen liefern. Über Palpation der Rippen sowie der Interkostalräume lässt sich eine hypomobile Rippe vergleichsweise einfach identifizieren (Schritt 1 – M).

**Figure 6:**
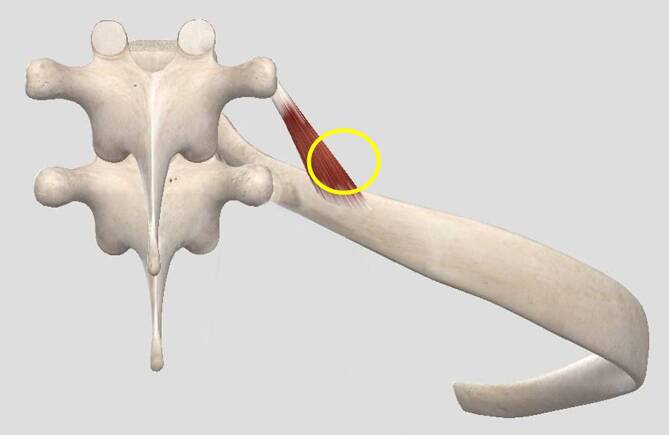

**I**rritation: Auch für die Rippenfunktion gibt es einen Irritationspunkt, morphologisches Korrelat ist der segmental zugehörige M. levator costae, knapp medial des Angulus costae am Oberrand der Rippe (Abb. 6) (Schritt 2 – I).

**P**rovocation: Im Provokationstest wird untersucht, wie sich der Irritationspunkt unter Palpation bei Inspiration und Exspiration verhält. Nehmen Tonus und Druckempfindlichkeit ab, so spricht man von der freien Richtung (Schritt 3 – P).

### Manuelle Therapie an Wirbelsäule und Rippen

Grundsätzlich können im thorakalen Bereich alle manualmedizinischen Techniken, wie sie im Artikel *Die segmentale und somatische Dysfunktion (H. Schnell et al.)* beschrieben wurden, angewandt werden. Die manuelle Behandlung von BWS-Wirbelsegmenten folgt dem gleichen Algorithmus wie an HWS und LWS, wenngleich die Grifftechniken der Anatomie angepasst sein müssen. Als weiteren besonderen Angriffspunkt bieten sich, wie auch in der Diagnostik, die Rippen an. Wegen der klaren segmentalen Zuordnung, der relativ oberflächlichen Lage und der guten Palpierbarkeit können in den zugehörigen artikulären Strukturen segmental gezielt propriozeptive Reize gesetzt werden [1, 3, 4].

### Zwerchfell und Viszera

Spezielle Optionen zu manueller Behandlung im Thoraxbereich bestehen über das Zwerchfell und die Viszera. Auch hier ist das Ziel, propriozeptive Reize gezielt in betroffene Segmente zu setzen sowie lokale Spannungen zu reduzieren.

Das Zwerchfell inseriert an der Vorderseite der Wirbel im thorakolumbalen Übergang, an den unteren Rippen sowie am Sternum. Erhöhte diaphragmale Spannung und damit Zug an Rippen und Wirbeln kann somit unmittelbar zu segmentalen Dysfunktionen führen. Darüber hinaus besteht eine enge anatomische Verbindung zum M. psoas und zum M. quadratus lumborum. Die beiden letzteren können im Sinne des Tensegrity-Modells Spannungsänderungen myofaszial nach kaudal übertragen [12].

Die Innervation des Zwerchfells erfolgt im Wesentlichen über den N. phrenicus, segmentaler Ursprung C 3–5. Nozizeptive und propriozeptive Reize am Zwerchfell werden primär in der mittleren Halswirbelsäule verschaltet und können demzufolge segmentale und somatische Dysfunktionen der mittleren HWS unterhalten bzw. auflösen.

Auch über Zwerchfell und Viscera können gezielt propriozeptive Reize segmental gesetzt werden

Die viszeralen Afferenzen der Thorax- und Oberbauchorgane folgen vielfach der efferenten Innervation entlang der Sympathikusfasern (s. oben). Nozizeptive Reize, aber auch propriozeptive Reize, die therapeutisch an den Organen gesetzt werden, gelangen auf diesem Weg zum spinothalamischen Projektionsneuron in thorakalen Segmenten und können hier zu segmentalen und somatischen Dysfunktionen führen oder diese auflösen. Zusätzlich gelangen viele Afferenzen über Fasern des N. vagus direkt in den Hirnstamm. Es resultiert ein hochkomplexes Netzwerk, dessen Kenntnis differenzialdiagnostisch und therapeutisch sehr hilfreich sein kann.

## Fallbeispiel 1

Etwa 3 Monate nach Knochenmarkkontusionen von BWK 4–7 (ohne Höhenminderung, im Verlauf keine Sinterung) stellt sich eine 49-Jährige mit folgender Beschwerdekonstellation vor:

- Schmerzpersistenz an der BWS
- Palpitationen und thorakale Enge, bereits mehrfache kardiologische Abklärung ohne wegweisenden Befund. Ergänzend pulmologische Abklärung mit Lungenfunktion und Bildgebung, ohne wegweisenden Befund
- Oberbauchbauchbeschwerden, Gastroskopie und Sonografie ohne pathologischen Befund

Multiple vorangegangene Arztkontakte und Erklärungen wie: „Sie haben nichts“, „das muss psychisch sein“ sowie „mit dem Unfall hat das nichts zu tun“ (D-Arztverfahren), zunehmende Frustration und Arztskepsis.

Die ergänzende manualmedizinische Untersuchung (nach MIP-Algorithmus) zeigt segmentale und somatische Dysfunktionen: 4. Rippe links (CG+4 insp), BWK6 und 7 (Th+6 rero-insp und Th7+ liro-insp).

### Therapie

Zunächst werden myofasziale Techniken im ehemaligen Frakturgebiet angewandt. Die Dysfunktionen der Rippengelenke und Wirbelgelenke werden dann mit spezifischen Grifftechniken behandelt.

### Verlauf

Bei der Kontrolluntersuchung nach 10 Tagen berichtet die Patientin über einen deutlichen Rückgang der Schmerzen, besseren Schlaf und keine Palpitationen mehr. Noch gelegentlich Sodbrennen. Nach zwei weiteren Behandlungen im Abstand von je ca. 2 Wochen ist und bleibt die Patientin beschwerdefrei.

### Diskussion

In den hier betroffenen Segmenten (Th4–Th7) liegen auch sympathische Ursprungsneurone, die mit an der Steuerung von Herz und Oberbauchorganen beteiligt sind (Abb. 2). Der positive Verlauf nach Auflösen der Dysfunktionen bestätigt die Arbeitshypothese einer vertebroviszeralen Verkettungsproblematik.

## Fallbeispiel 2

Ein 35-jähriger Patient stellt sich mit starken Schmerzen (seit 3 Tagen) in der linken Schulter vor. Kein Trauma, kein vorangegangenes besonderes Training. Patient vorbekannt nach VKB-Ruptur vor 5 Jahren und immer wieder Kniebeschwerden. Sonst gesund, ambitionierter Kraftsportler (190 cm, 95 kg, Körperfettanteil < 20 %).

### Diagnostik

#### Klinische Untersuchung

Schulter global frei beweglich, keine Schmerzprovokation möglich, Rotatorenmanschettentests negativ und von kräftiger Funktion in allen Ebenen, Hyperadduktionstest negativ, O’Brien-Test negativ, keine Druckschmerzen provozierbar, periphere Durchblutung, Motorik, Sensibilität ohne pathologischen Befund, HWS frei beweglich.

#### Sonografie

Rotatorenmanschette intakt, lange Bizepssehne mit unauffälligem Echosignal im Sulkus ohne Halo und ohne Luxationstendenz, diskrete Zeichen einer Bursitis subdeltoidea.

#### Klinischer Aspekt des Patienten

Patienten wirkt „irgendwie nicht gesund“ – nochmalige Nachfrage nach dem Allgemeinbefinden, Antwort: „besch…“. Auf Aufforderung tiefes Ein- und Ausatmen, dabei deutliche Schmerzzunahme. Bei Auskultation symmetrisches vesikuläres Atemgeräusch.

#### Manualmedizinischer Befund (nach MIP-Algorithmus)

Rippendysfunktion der ersten und dritten Rippe links.

### Therapie

Entscheidung zum Worst-Case-Prozedere: praxisinternes Notfallprozedere mit Maßnahmen siehe Abb. 1, Notfalleinweisung per Rettungsdienst mit Notarzt in nächste Chest-Pain-Unit unter der Verdachtsdiagnose Lungenarterienembolie.

### Diagnose

Beidseitige fulminante Lungenarterienembolie, mutmaßlich im Kontext mit Steroidabusus.

### Verlauf

Komplikationsloser Verlauf mit folgender Antikoagulation.

### Diskussion

Die Schwierigkeit in diesem Fall war, „daran zu denken“, der sehr sportliche Mann konnte auch eine massive Reduktion der Lungenfunktion für Alltagsbelastungen wie z. B. Treppe gehen noch sehr gut kompensieren. Der entscheidende klinische Aspekt war die starke Schmerzunahme bei tiefer Inspiration, gepaart mit dem ungesunden Gesichtsausdruck. Atemabhängige Schmerzen können gleichzeitig aber auch bei segmentalen und somatischen Dysfunktionen an BWS und Rippen auftreten, in diesem Fall hat die Lungenarterienembolie auch zu reflektorischen Dysfunktionen der Rippen 1 und 3 auf der linken Seite geführt, die mittels MIP-Diagnostik ermittelt werden konnten. Priorität hat in diesem Fall natürlich die auslösende Pathologie.

Die zweite Herausforderung war, dass das Worst-Case-Prozedere auch gegen Widerstände durchgesetzt werden musst. Der Patient wollte zunächst nicht in die Klinik – und wenn schon, dann selbst mit dem Auto fahren. Rettungsdienst und Notarzt waren skeptisch ob der Verdachtsdiagnose, die Dienstärztin im Telefonat zur Anmeldung: „wie sind denn die D‑Dimere?“ und auf den Hinweis, dass sich der Patient in einer orthopädischen Praxis befindet folgte: „ja ja, schicken Sie halt …“

### Fazit

Die Lungenarterienembolie ist und bleibt ein Chamäleon, auch bei Frauen mit hormoneller Kontrazeption und Nikotinabusus sowie natürlich im Rahmen von Ruhigstellungen kommt es gehäuft zu thromboembolischen Ereignissen. Fehlender Wadenschmerz beweist gar nichts. In dubio pro worst-case!

## Zusammenfassung

Einige der möglichen Differenzialdiagnosen bei Thoraxschmerz sind potenziell akut lebensbedrohlich und müssen konsequent einer adäquaten Notfalldiagnostik und -therapie zugeführt werden. Das Auffinden und Therapieren segmentaler und somatischer Dysfunktionen am thorakalen Achsenskelett kann für Patienten nach mehrfacher Ausschlussdiagnostik ohne wegweisenden Befund auf kardiologischer, pulmologischer und abdomineller Ebene äußerst befreiend sein, nicht zuletzt, weil sonst häufig nur das Etikett „psychische Genese“ bleibt.

## Fazit für die Praxis

- Segmentale und somatische Dysfunktionen thorakaler Segmente können vielfältige Symptome auslösen, da hier das somatische Nervensystem eng mit dem vegetativen Nervensystem vernetzt ist.
- „Unspezifisch“ anmutende thorakale und viszerale Symptome können ihren Ursprung in einer segmentalen und somatischen Dysfunktion der thorakalen Segmente haben.
- Bei rezidivierender segmentaler und somatischer Dysfunktion im immer gleichen Segment muss an eine (beginnende) Erkrankung im zugehörigen Viszerotom gedacht und entsprechend danach gefahndet werden.
- Das Leitsymptom „Thoraxschmerz“ mit den lebensbedrohlichen Differenzialdiagnosen hat immer Priorität und muss im Zweifelsfall zu interdisziplinärer Notfalldiagnostik führen.
- Auch eine orthopädisch-unfallchirurgische Praxis muss für Notfälle gerüstet sein. Dazu zählen: regelmäßiges Notfalltraining des gesamten Teams, definiertes Notfallprozedere und adäquate technische Ausstattung.
- Manuelle Medizin ist differenzialdiagnostisch wertvoll. Die manuelle Therapie von segmentalen und somatischen Dysfunktionen kann zu eindrucksvollen Ergebnissen führen.

## Abkürzungen

BWK: Brustwirbelkörper
BWS: Brustwirbelsäule
HWS: Halswirbelsäule
LWS: Lendenwirbelsäule
MIP: Mobility, Irritation, Provocation
VKB: Vorderes Kreuzband
